# Evaluation of cannabidiol-based products in Brazil: how can current regulations influence their labeling quality?

**DOI:** 10.1186/s42238-025-00270-2

**Published:** 2025-02-22

**Authors:** Andrea Donatti Gallassi, André Wagner Carvalho de Oliveira, Nathália Silva Mendes, Renato Filev, Eduardo Yoshio Nakano

**Affiliations:** 1 Graduate Program in Health Sciences and Technology (Programa de Pós-Graduação em Ciências e Tecnologias em Saúde), Faculdade de Ciências e Tecnologias em Saúde (FCTS), Universidade de Brasília (UnB), Centro Metropolitano 1, Conjunto A, Ceilândia Sul, ZIP 72220-900 Brasília-DF, Brasil; 2 Center of Drugs and Associated Vulnerabilities (Centro de Referência sobre Drogas e Vulnerabilidades Associadas), FCTS, UnB, Brasília, DF Brazil; 3 Orientation and Assistance Program for Dependents (Programa de Orientação e Atendimento a Dependentes – PROAD), Universidade Federal de São Paulo (UNIFESP), São Paulo, SP Brazil; 4 Statistical Department, (Departamento de Estatística), UnB, Darcy Ribeiro Campus, Brasília, DF Brazil

**Keywords:** Cannabidiol (CBD), Labeling quality score, Safety use, Regulation, Brazil

## Abstract

There is concern about the quality of cannabis-based products used in Brazil, mainly cannabidiol (CBD). This study aimed to evaluate the quality of labeling on CBD products marketed in Brazil authorized by two regulations – N660/2022 on imported products and N327/2019 on products with temporary trade permits – and whether there were differences between them concerning four domains: prescription, good manufacturing practices (GMP), laboratory tests, and safety of use. Determined labeling quality was based on a score of 45 criteria divided per domain and weights from 1 to 3 (according to the relevance for users' and prescribers' safety) built by public information provided by product manufacturers/representatives on websites and e-mail consultations. The quality score was classified as very satisfactory, satisfactory, or not very satisfactory, represented in median and interquartile range. Between the N327 and N660 products, the quality scores were compared using the Mann–Whitney U-test. All tests considered two-tailed hypotheses and a significance level of 5%. After applying the inclusion criteria, from 148 products selected, 105 were evaluated. Most of the evaluated CBD products were classified as satisfactory (47), followed by not very satisfactory (39) and very satisfactory (19). The N327 products presented information that was more accessible than the N660 products. Similarly, there was a significant difference between the two groups concerning the domains of prescription and safety of use; products in the N327 showed better results than those in the N660. This study contributes to the urgent debate on the quality of labeling and the risks of CBD widely marketed in Brazil.

**Supplementary Information:**

The online version contains supplementary material available at 10.1186/s42238-025-00270-2.

## Highlights

• Creation of a quality score to evaluate cannabidiol (CBD)-based product labeling practices.

• Pioneering comparison of the quality of CBD authorized by Brazil's two regulations.

• Valuable guide to CBD labeling quality for prescribers and patients.

• Important information on CBD labeling quality to support decisions from regulatory agencies.

• Urgent discussion on the importance of labeling regulations for the quality of CBD marketed.

**Supplementary Information:**

The online version contains supplementary material available at 10.1186/s42238-025-00270-2.

## Introduction

*Cannabis* spp. has been used for therapeutic purposes for approximately 5000 years (Bonini et al., 2018). This ancient plant has a long history of use in the treatment of various diseases (Rodrigues et al. 2020; Zuardi, 2006).

Recently, a growing number of scientific studies have demonstrated the therapeutic potential of various compounds from this plant to treat or alleviate symptoms of various health problems, such as neurodevelopmental and neurodegenerative disorders, autoimmune diseases, cancers, human immunodeficiency virus (HIV), mental disorders and substance use disorders (Andre et al. 2016; Rodrigues et al. 2020; Whiting et al. 2015; Zuardi et al., 2006; Gallassi et al. 2024). In this sense, it is possible to consider that there is a satisfactory degree of evidence for the use of cannabis compounds for epilepsy (especially cannabidiol (CBD); Lattanzi et al. 2021), for chronic/neuropathic pain (with high proportions of delta-9 tetrahydrocannabinol (∆9-THC); McDonagh et al. 2022), and for spasms due to multiple sclerosis (Filippini et al. 2022). Although there is possible therapeutic potential for cannabis as a treatment for other conditions, such as adverse events from cancer chemotherapy, including nausea, vomiting and loss of appetite (Bathula & Maciver 2024), symptoms of autism spectrum disorder (Holdman et al. 2022), Parkinson's disease (Urbi et al. 2022), Alzheimer's disease (Lim et al. 2017), fibromyalgia (Strand et al. 2023), anxiety disorders (Black et al., 2019), post-traumatic stress disorder (Orsolini et al. 2019), and substance use disorders (Gallassi et al. 2024; Rodrigues et al. 2020), the data is still moderate, insufficient, or inconclusive, requiring further studies to support the safety, efficacy and feasibility of its clinical application.

Considering the therapeutic potential of cannabis, the total or partial regulation of the plant's production chain in several countries has enabled access to the use of cannabis and a greater volume of research to understand its effects and therapeutic potential (Santos et al., 2019). However, the legalization, regulation, implementation and application of cannabis use are different processes between countries, and these differences can affect the results of the policy of access to these treatments. With many changes in a short time in the cannabis legalization landscape worldwide, it is crucial to evaluate ongoing legislation and policies and proceed with evidence-based policies and practices (Johnson et al., 2023). The prohibitionist policy in the USA, as in other countries, has directed research towards the harm caused by the social use of the plant, maintaining gaps in scientific knowledge about the therapeutic potential of cannabis. There is also the paradox of the balance between the strong public demand for reform of the policy on access to cannabis products and the current lack of scientific consensus on therapeutic indications (Schauuer et al., 2023).

It is estimated that around 6.9 million patients in Brazil could be treated with cannabis-derived products, making it imperative to constantly update laws and regulations to facilitate access to high-quality products, both for research and therapeutic purposes (Ministry of Health 2019). The loosening of regulatory oversight of hemp in the USA, for example, has resulted in the marketing and sale of CBD-based products with questionable ingredients and quality (Johnson et al. 2022). There is concern from the academic community about the chemical compounds in unregulated CBD products, including fungal contamination, harmful by-products of the manufacturing process and the presence of other synthetic or semi-synthetic psychoactive substances (JWH compounds, cathinones, ∆8-THC; FDA May 31, 2019). These points reflect the need to ensure the quality of cannabis-based products, which have a direct impact on user safety (Johnson et al. 2022).

In Brazil, the exponential growth in the use of cannabis-based products for therapeutic purposes was driven by the relaxation of Brazilian regulations since 2015. Due to a solid social mobilization, CBD was included in the list of substances subject to special control by the National Health Surveillance Agency (ANVISA; Ministry of Health 1998) and was no longer on the list of proscribed substances, being prescribed as a medication. Patients could import it based on a prescription from a legally authorized provider (Ministry of Health 2015).

Brazilian legislation only partially allows the national production of cannabis-based products for medical and research purposes (Brasil 2006). However, this supposed permission has never been authorized by the competent authority—the Ministry of Health—and the current cultivation in the country (domestic and associative) is supported only by court decisions. In this sense, ANVISA regulations allow access through (i) importation for personal use (main means used for purchase), with medical prescription and authorization from ANVISA itself (RDC 660/2022, here referred to as N660—"normative"; Ministry of Health 2022a, b); and (ii) the acquisition of products with temporary trade permits in pharmacies upon presentation of a medical prescription (RDC 327/2019 – N327; Ministry of Health 2019).

The number of imported products authorized by ANVISA (N660) is much larger than those notified with temporary trade permits in Brazil (N327). However, even the notified products have not yet been assessed by ANVISA for their efficacy or safety, and the temporary trade permit was exceptionally approved (ANVISA, 2024). The temporary authorization was granted at the request of the companies and is valid for five years (2019–2024). The companies undertook safety and efficacy studies in order to apply for final product registration once the temporary permit expired.

ANVISA reports that in 2022, 79,993 applications to import cannabis-derived products were granted, almost double the number from the previous year (ANVISA, 2022). This wide availability of cannabis-based products and the means of accessing them, supported by a weak legal system, quality control and inspection, can lead to significant safety risks and potential damage to the treatment of the health problems for which it is indicated, given the heterogeneous composition and quality of the products, including their poor labeling.

In this sense, this study aimed to present and discuss the quality of labeling on CBD-based products marketed in Brazil that are included in the two ANVISA regulations – N660/2022 on imported products and N327/2019 on products with temporary trade permits – based on the public information available by manufacturers/representatives on websites and/or by e-mail consultations*.* The research was designed to verify differences in labeling practices between the two product groups based on the publicly available information and whether there were differences between them in the domains established by the study, called prescription, good manufacturing practices (GMP), laboratory tests (certificate of laboratory analysis – CoA) and safety of use.

## Materials and methods

### Research stages

This study evaluated the quality of labeling of CBD-based products marketed in Brazil. It presents a quality score based on public information provided by product manufacturers/representatives on websites and through e-mail consultation. The products were not evaluated on site, as most of the products consumed in Brazil are purchased online. This is because only those with temporary trade permits are allowed to sell in pharmacies, making them a smaller and more expensive proportion of the total compared to imported products. In other words, prescribers and patients rely on the information available on the websites of the product manufacturers/representatives.

The study was designed in three stages. The first was based on defining the composition of the cannabidiol-based products to be considered. Products with different compositions were considered – rich in CBD in full spectrum, broad-spectrum, or isolated modalities (THC free), giving preference to the full spectrum when there was more than one type of qualitative and quantitative composition of the same product – for oral use with any possible pharmaceutical presentation (oil, capsule, tablet, oral spray), and which were notified, registered, imported and/or sold via ANVISA authorization until January 2023. In the second stage, the Brazilian regulations and those countries that allow the medical use of cannabis (the United States, Israel, Uruguay, the Netherlands, Canada, Portugal, Australia, Colombia, Chile, England and China) were studied. This phase aimed to verify whether these countries have any regulations/norms on labeling and quality criteria for commercialized cannabis-based products. The third and final stage was a literature review of studies published in English in the leading medical and health-related databases, such as Medline/PubMed, Embase, PsycINFO, and Scielo, using the descriptors "medicinal cannabis OR medical cannabis OR cannabidiol or CBD" AND "quality control", to identify, according to the scientific literature, which criteria are considered relevant to the quality and safety of the cannabis-based products.

The three stages of the study served as a basis for defining the labeling quality criteria used to build a quality score, in which each product considered in this study was evaluated and classified. The products selected for evaluation and classification according to the quality score were based on ANVISA regulations N660/2022 (unregistered products imported at least once; Table S1 – Supplementary Material) and N327/2019 (notified products with temporary trade permits). In addition, the two most significant marketplaces for CBD-based products operating in Brazil were consulted to check the compatibility of the products set out in N660 and N327 and their availability for purchase. These two sales platforms for cannabis-based products were selected considering their wide availability of products (diversity), including the largest number of products listed in the two regulations, especially N660, which is not available for sale in pharmacies. In addition, both platforms supplied the entire country (coverage), and they are best known by prescribers and patients seeking information about cannabis-based products that could be used in their treatments.

The selection of the N660 and N327 products evaluated was based on the following criteria: (i) having labeling information for public consultation on the manufacturer's or representative's websites; (ii) providing some means of contact to request additional labeling information that was not publicly available (including the CoA); (iii) be available for sale on the two largest marketplaces operating in Brazil. These criteria were established to evaluate the most accessible products for prescribers and patients, both in terms of technical information and purchase. The products listed in N660 are those that have already been imported into Brazil even once, which means that the list is constantly updated. In this sense, the study focused on evaluating the most widely used products in the country.

### Definition of quality criteria

Based on the three initial stages of the study, 45 criteria were defined to build the labeling quality score. When provided, the information needed to meet the quality criteria specified in the study had to be present on the label, primary or secondary packaging, package insert or information leaflet, or CoA of the products.

To compile the score, weights from 1 to 3 were set according to the quality criteria (Table S2 – Supplementary Material). A weight of 3 was given to items that were considered very important for the prescription and clinical indication of the products and, consequently, for the safety of users and prescribers (i.e., the concentration of each active ingredient and criteria for matching the number of drops and mg of the main phytocannabinoids). A weight of 2 was given to the specific quality criteria of products that were considered not essential for their prescription and clinical indication, but that help promote the safety of use by patients (e.g., the way the product is used and storage precautions). Complementary labeling information not essential for prescription, clinical indication, or safe use (e.g., safe disposal and the physical and organoleptic characteristics of the product) was given a weight of 1.

All evaluated products were scored according to the availability of information for public access via product labels, packaging, or inserts, online publication, and/or direct contact by e-mail with the company/manufacturer, generating a result that refers to the score achieved by the products in the four domains after the evaluation.

### Definition of domains

The quality criteria were divided into four domains to compare the labeling of the N660 and N327 products (Table S2). The first domain was prescription, in which items essential for the clinical indication, prescription, and dispensing of CBD-based products, with specifications related to their qualitative and quantitative aspects, were selected. The second domain was good manufacturing practices (GMP), with criteria fundamental to ensuring product quality before it reaches the user. The third domain dealt with safety of use, i.e., all the criteria usually included in the package leaflet or information leaflet that guarantee the appropriate mode of use and possible risks to the users. The last domain concerns laboratory testing (CoA), which includes the criteria for laboratory analysis of products regarding their qualitative and quantitative content of phytocannabinoids, terpenes, and the absence of contaminants, such as heavy metals, mycotoxins/anaphylatoxins, and pathogen colony-forming units (CFU).

### Contacting manufacturers or representatives

**Fig. 1:**
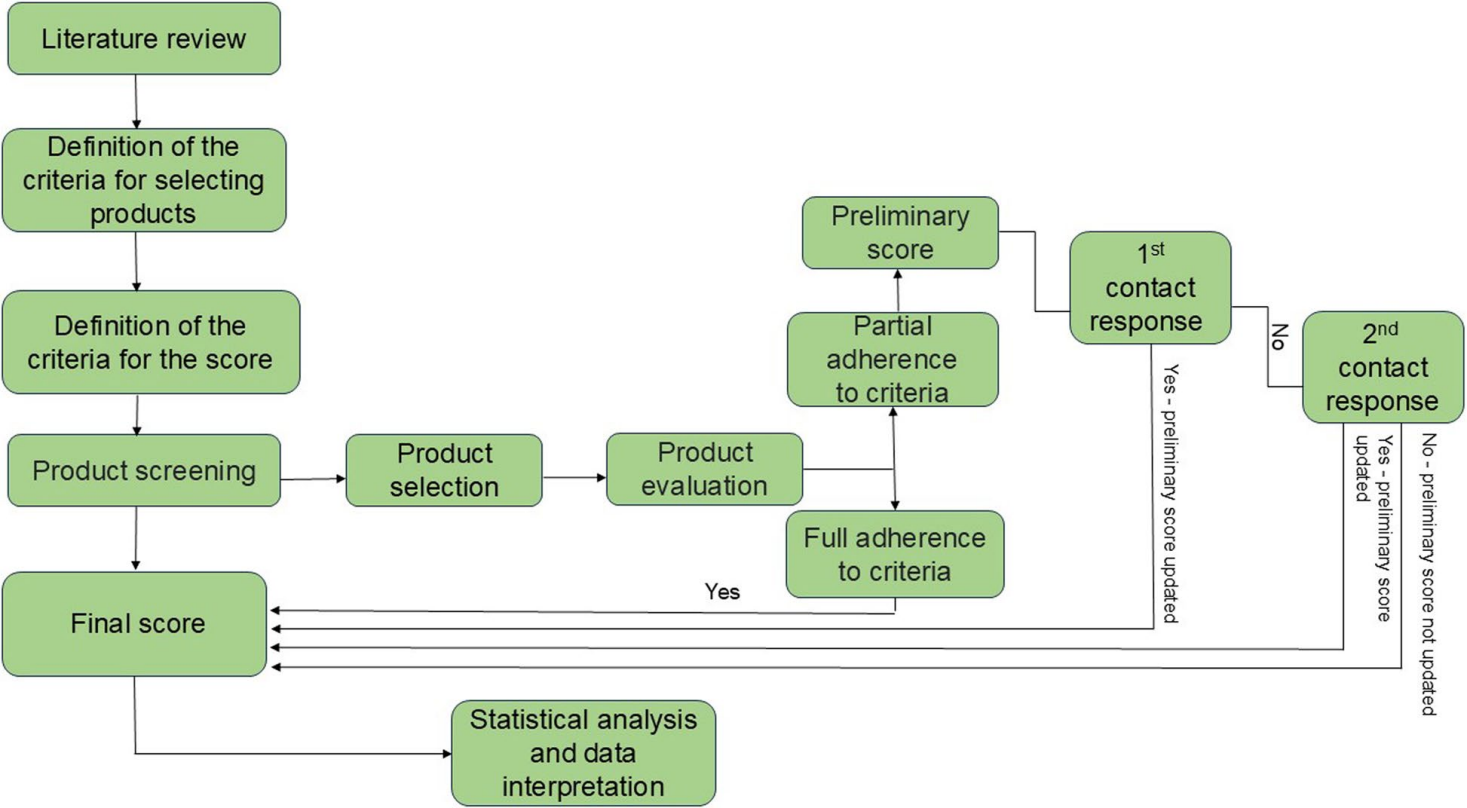
Flowchart of the research stages

When all the quality criteria established by the study were not identified through online consultations on the manufacturers'/representatives' websites and marketplaces (on the product's labeling, packaging, package leaflet, information leaflet, or CoA), emails were sent requesting the missing information. If a response was not received within 14 days, a new e-mail was sent with a seven-day response deadline (Fig. 1).

### Classification of scores

According to the e-mail replies, the products' labeling quality score could increase or remain unchanged. No conflicting information existed between labels, websites and email responses, so this did not impact the products’ scores. As a result, after the first evaluation, the products received a preliminary quality score. They received a final quality score after receiving or not receiving the missing information in answer to the e-mail. Depending on the product's final quality score, it could be classified as very satisfactory (50 points or more), satisfactory (25–49 points), or not very satisfactory (0–24 points).

### Statistical analysis

The total quality score and its four domains were represented in terms of the median and interquartile range (IQR; P25-P75). Due to the lack of normality of most of the samples (verified by Shapiro–Wilk test) the quality scores between the N327 and N660 products were compared using the Mann–Whitney U-test. All tests considered two-tailed hypotheses and a significance level of 5%.

## Results

**Fig. 2:**
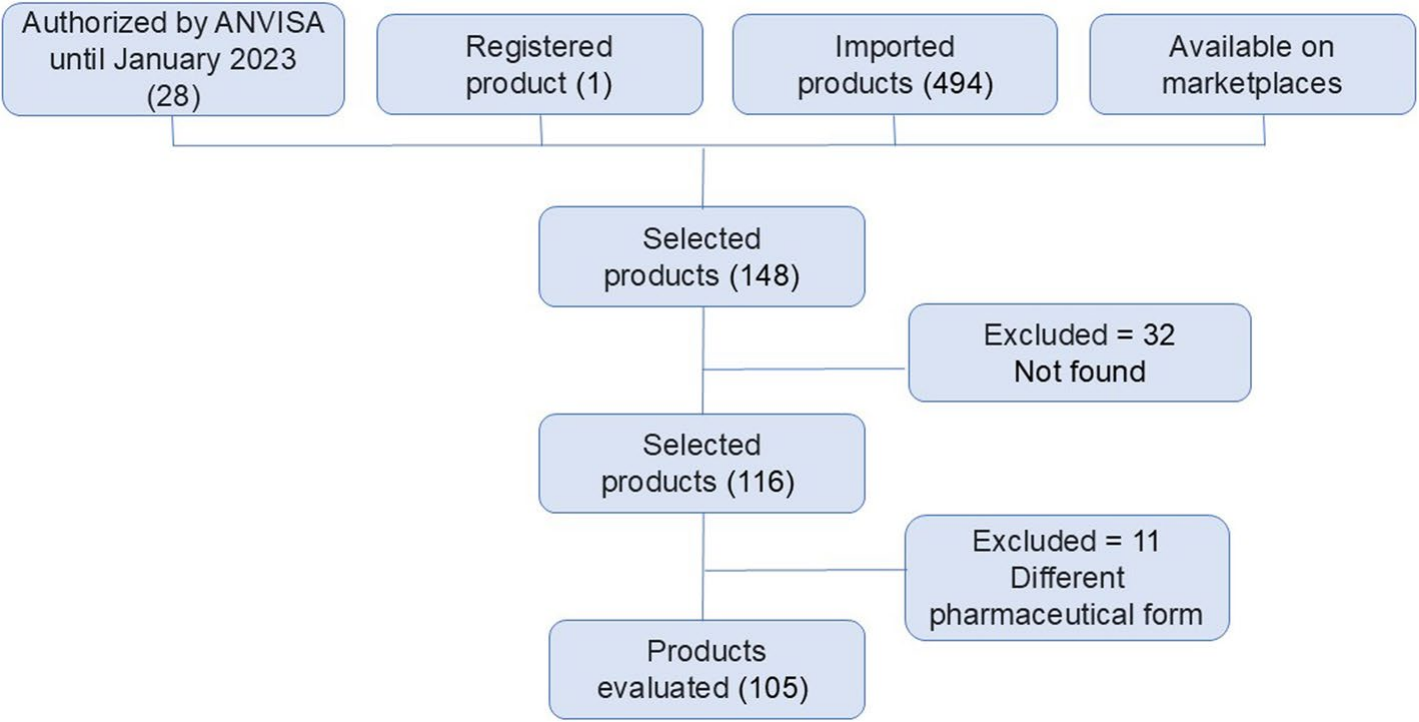
Flowchart of the steps for including products on the final list

A total of 148 products were selected. During the product information search stage, 32 products could not be found on the manufacturers' or representatives' websites; for example, some products did not contain the information required for the initial selection. In addition, 11 products were excluded from the list because they did not meet the pharmaceutical form of interest for the study (oral administration). After reviewing the inclusion criteria, the final list included 105 evaluated products (Fig. 2; Table 1). Mevatyl®, which has a higher proportion of THC (27 mg/ml) than CBD (25 mg/ml), was selected because it is the only cannabis-based product to have been definitively registered with ANVISA and is, therefore, part of N327.

**Table 1:** List of the 105 products evaluated presented in descending order according to their final labeling quality score

Product	Manufacturer	Normative	Product type	Preliminary Score	Final Score
**Products with a very satisfactory score**					
CBfarma—Espectro Completo—CBD 500 mg MCT (CBD 500 mg)/frasco 30 ml	CBFarma	N660	Oil	52	68
Nunature Labs—Espectro Completo—Canabidiol NuNature Oil (34,36 mg/ml)/frasco 30 ml	Nunature Labs	N327	Oil	65	65
Extrato de Cannabis sativa Herbarium 43 mg/mL	Herbarium	N327	Oil	62	62
Alliant CBD	Alliant	N660	Oil	62	62
USAHemp CBD full spectrum	USA Hemp CBD	N660	Oil	41	62
Alma CBD	CaniBrands Inc	N660	Oil	61	61
Canabidiol Greencare 23,75 mg/ml	Greencare	N327	Extract	59	59
Extrato de Cannabis sativa Cann 10 Pharma 200 mg/ml	Cann 10 Pharma	N327	Extract	59	59
Canabidiol Mantecorp Farmasa 23,75 mg/ml	Mantecorp Farmasa	N327	Oil	59	59
Lazarus Naturals—Espectro Completo—Sleep + Melatonina Capsulas—(CBD 30 mg + CBG 10 mg + CBN 10 mg por cápsula)/frasco 40 cps	Lazarus Naturals	N660	Capsule	57	57
Canabidiol Farmanguinhos	Farmanguinhos	N327	Oil	56	56
Epidiolex	GW Pharmaceuticals	N660	Oil	56	56
Belcher—Isolado—Canabidiol Belcher—(CBD 150 mg/ml)/frasco 10 ml	Belcher	N327	Oil	55	55
Canabidiol Aura Pharma	Aura Pharma	N327	Oil	52	52
Canabidiol Active Pharmaceutica 20 mg/ml	Active Caldic	N327	Oil	52	52
Mevatyl	Ipsen	N327	Spray	50	50
MEDTerra's CBD Oil	MEDTerra	N660	Oil	50	50
Carmen’s Medicinals Full Spectrum Cannabinoids	Carmen’s Medicinals Full Spectrum	N660	Oil	50	50
Elixinol—Espectro Amplo—Everyday Rapid Reset Liposome (CBD 1000 mg)/frasco 100 ml	Elixinol LCC	N660	Oil	50	50
**Products with a satisfactory score**					
Alese CBD	Korasana	N660	Oil	49	49
Epixann 10%	Caillon Hamonet	N660	Oil	29	46
Eliv—Espectro Completo—CBD Starter—1500 mg CBD—30 ml	Korasana	N660	Oil	45	45
Canna River—Isolado—Delta 8 Tincture (1000 mg)/frasco 15 ml	Canna River LCC	N660	Tincture	45	45
Beyond Botanicals	Beyond Botanicals LLC	N660	Oil	45	45
Neurogan CBD	Neurogan	N660	Oil	43	43
Enecta CBD	Enecta	N660	Oil	32	43
Canabidiol Collect (20 mg/ml)	Collect	N327	Oil	42	42
ECS Care	ECS Therapeutics LLC	N660	Oil	42	42
Organic CBD Oil	Hempen Organic	N660	Oil	42	42
Óleo CBD full spectrum—6000 mg	USA Hemp CBD	N660	Oil	41	41
Verdemed CBD	Verdemed	N327	Oil	41	41
Nuleaf Naturals CBD Oil	Nuleaf Naturals LLC	N660	Oil	41	41
Cibdol	Cibdol bv	N660	Oil	40	40
Prati-Donaduzzi—Isolado—Óleo de CBD 200 mg/ml (CBD 6000 mg)/frasco 30 ml	Prati-Donaduzzi	N327	Oil	40	40
HempMeds—Espectro Competo—RSHO-BR—CBD Oil (CBD 3000 mg)/frasco 30 ml	Hemp Meds Px	N660	Oil	39	39
Hemp & Olive	Green Gorilla	N660	Oil	39	39
NanoLab CBD	NanoLab Nutrition LLC	N660	Oil	38	38
Tegra EUROLINE CBD	Korasana	N660	Oil	38	38
Endoca Hemp Oil	Endoca	N660	Oil	37	37
Nordic Oil CBD	Nordic Health Group	N660	Oil	36	36
Charlotte Web Hemp Extract	CW Botanicals	N660	Extract	36	36
CBDAlchemy Oil	CBDAlchemy	N660	Oil	26	36
FAB CBD	FAB Nutrition	N660	Oil	35	35
Alivitta CBD	ALIVITTA LLC	N660	Oil	35	35
Green Monkey CBD Oil 1500 mg	Green Monkey CBD Store	N660	Oil	35	35
Cibadol Cannabidiol	Cibadol	N660	Oil	35	35
Allandiol CBD	Biocase Brasil	N660	Oil	34	34
Leaf CBD	Leafmed Care	N660	Oil	32	32
Evona CBD	Hemp For Fitness LLC	N660	Oil	32	32
Valenss Wellness CBD	Valenss Wellness	N660	Oil	32	32
Provacan CBD	Ciitech	N660	Oil	32	32
Isospec—Auttrum—Espectro Completo—Oil (CBD 1500 mg + CBG 1500 mg)/frasco 30 ml	Isospec Ltd	N660	Oil	31	31
Golden CBD—Isolado—Slim THCV 500 mg (THCV 500 mg)/frasco 30 ml	Golden CBD	N660	Oil	31	31
CannaBrasil	CannaBrasil	N327	Extract	31	31
Cannamedic CBD Oil	Cannamedic B.V	N660	Oil	30	30
CBD Vida—Nano-infused CBD	CBD Vida	N660	Oil	29	29
Entourage CBD	The Native Hemp	N660	Oil	29	29
MGC Pharma (CBD/THC)	MGC Pharma	N660	Oil	28	28
LGP Classic	Little Green Pharma	N660	Oil	27	27
ELC CBD	Ease Labs	N327	Oil	27	27
Verdecann Aceite CBD	Verdecann	N660	Oil	26	26
EVR Hemp Oil CBD	EVR Premium Hemp Oil	N660	Oil	26	26
Epifractan CBD (5%)	Medicplast S/A	N660	Oil	26	26
Sativida CBD	Sativida	N660	Oil	25	25
Dixie Botanicals	Hemp Oil Hemp Meds Px	N660	Oil	25	25
Cibdex Hemp CBD Complex	Hemp Meds Px	N660	Oil	25	25
**Products with a not very satisfactory score**					
EcoGen CBD	EcoGen Laboratories	N660	Oil	24	24
Tilray	Tilray Medical	N660	Oil	23	23
Delta 8 CBD—Delta 8 Pharma Grade	Delta 8 Pharma Grade	N660	Oil	23	23
Hempflex Full 3000 mg	Green Care	N660	Oil	23	23
CBD Calm	Kemin Industries Inc	N660	Oil	22	22
Fern Valley Farms	Fern Valley Farms	N660	Oil	22	22
Healist Naturals CBD	Healist Advanced Naturals LLC	N660	Oil	21	21
Carolina CBD	Carolina CBD Solutions	N660	Oil	20	20
CBD Emporium	CBD Emporium	N660	Oil	20	20
Bisaliv CBD	Thronus Medical INC	N660	Oil	19	19
Feel Good Health CBD—Feel Good Health	Feel Good Health	N660	Oil	19	19
Mahara CBD Oil	Mahara CBD Group	N660	Oil	19	19
DiolPure CBD	DiolPure	N660	Oil	19	19
Fitosil CBD—Fitosil	Fitosil	N660	Oil	18	18
Medcan Australia CBD	Medcan Australia	N660	Oil	18	18
Just Hemp CBD—Just Hemp	Just Hemp	N660	Oil	18	18
Nabix	Biota Biosciences	N660	Oil	18	18
Elixir Organic CBD	Elixir Organic	N660	Oil	18	18
Tinkun CBD	Tinkun Olam	N660	Oil	17	17
Medropharm CBD	Medropharm GmbH	N660	Oil	17	17
1 Pure CBD	Pure	N660	Oil	16	16
Isodiolex CBD (Isodiol)—Isodiol	Isodiol	N660	Oil	16	16
Clever Leaves CBD	Clever Leaves 360	N660	Oil	16	16
FoliuMed CBD—FoliuMed	FoliuMed	N660	Oil	15	15
Formula Swiss—Full Spectrum CBD—Formula Swiss A.G	Formula Swiss A.G	N660	Oil	15	15
Spectrum Therapeutics	Spectrum Therapeutics	N660	Oil	15	15
HempFlex CBD—Green Care—Green Care	Green Care	N660	Oil	14	14
FitoCBD—FitoFarma—Neurogan	FitoFarma	N660	Oil	13	13
Cannapresso CBD Oil—Tincture	Cannapresso CBD	N660	Tincture	13	13
Panaxia CBD/THC	Panaxia Pharmaceutical Industries Ltd	N660	Oil	12	12
Tegra USALINE CBD	Korasana	N660	Oil	12	12
Cannabin Omega	PBG Global	N660	Oil	11	11
Valens CBD	Valens CBD LLC	N660	Oil	11	11
Nuvita CBD Oil	Nuvita	N660	Oil	11	11
Tegra Latam Line	FoliuMed	N660	Oil	11	11
Blue Ridge Hemp CBD	Blue Ridge Hemp	N660	Oil	10	10
MedReleaf	MedRealef	N660	Oil	9	9
Greenmed—CBD—Greenmed	Greenmed	N660	Oil	9	9
CIDCAM CBD Aceite	CIDCAM Cannabis	N660	Oil	8	8

Table 1 shows the evaluation results of each product according to the labeling quality criteria established by the study. Of the 105 products evaluated, 19 were classified as very satisfactory, 47 as satisfactory, and 39 as not very satisfactory. The median quality score for very satisfactory products was 57.0 (IQR: 12.0–19.0), for satisfactory products 35.0 (IQR: 30.0–41.0), and for not very satisfactory products 16.0 (IQR: 12.0–17.0).

All 105 products on the list did not meet all of the labeling quality criteria considered in the study that are publicly available on their websites or marketplaces. Therefore, all the companies were contacted via e-mail, asking them to send the missing information. After sending the e-mails and ensuring that the response deadline was met, 12 responses were obtained, and 5 products increased their final quality score by providing some or all of the information requested (Table 1).

**Fig. 3:**
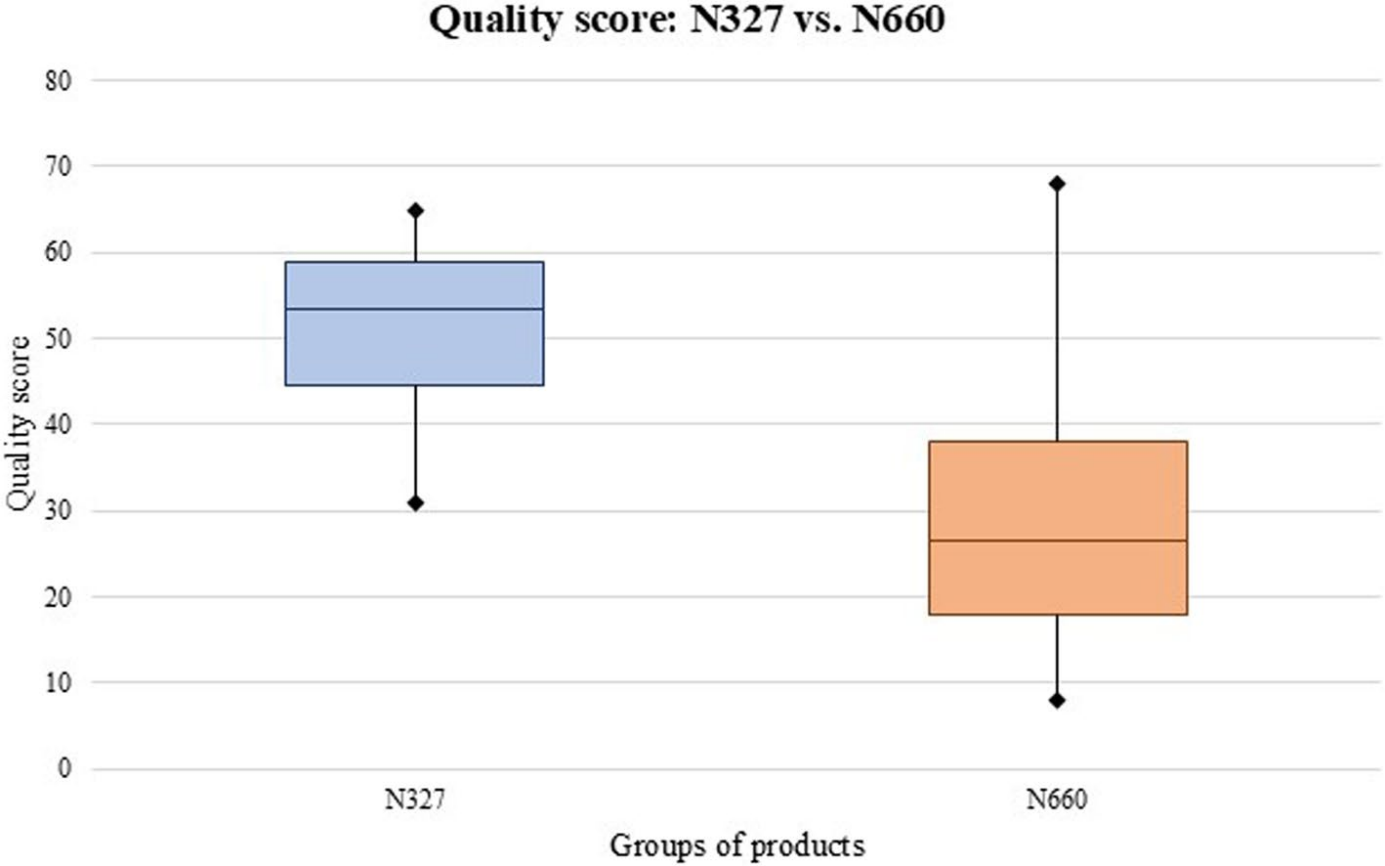
Comparison of the median labeling quality scores of the N327 group vs. those of the N660 groyup

To compare the public availability of information between the N660 and N327 products, an analysis was carried out between the medians of the products. The N327 group had a median score of 53.50 points (IQR: 41.75–59.00). The N660 group had a median of 26.50 points (IQR: 18.0–38.25). A statistically significant difference was found when the two product groups were compared (*p* < 0.001; Fig. 3).

Across all products, it was observed that manufacturers or representatives provided little essential information for patient safety. For example, only 40 products presented the CoA, 27 products described information on the correspondence between the number of drops and the milligrams of the main cannabinoids (1 drop = "X" mg CBD and/or THC), and 36 products presented information on contraindications, precautions and instructions for use. These were just a few examples of data that could have confounded the final score. Therefore, to understand the differences between the N327 and N660 groups, the items scored in the labeling quality score were divided into four domains, as explained above.

**Fig. 4:**
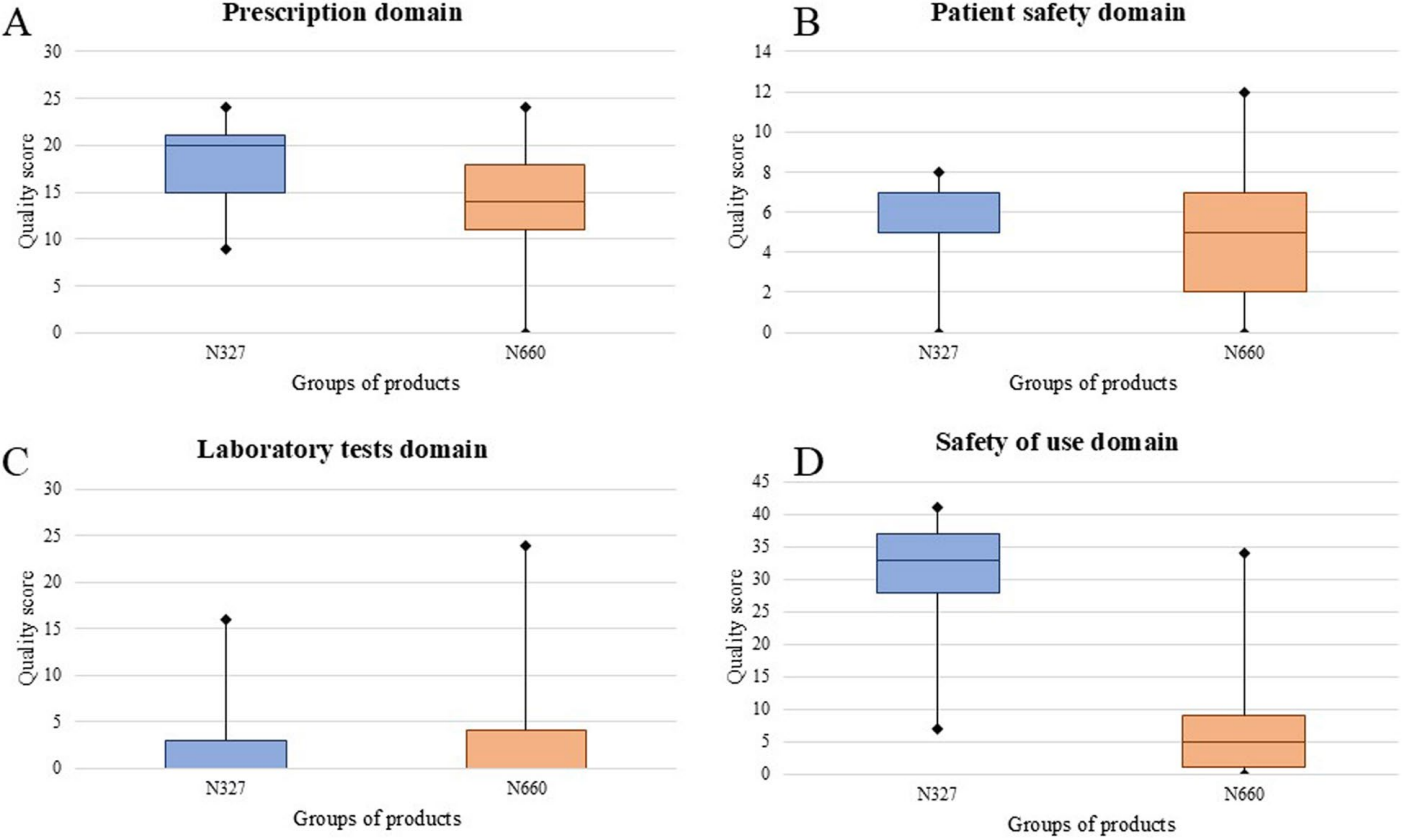
Comparison of the median labeling quality scores of the products in the N327 group vs. those in the N660 group according to the quality domain. **A** prescription domain; **B** good manufacturing practices (GMP) domain; **C** safety of use domain; and **D** laboratoty testing domain

In the prescribing domain, the N327 group had a median score of 20.0 (IQR: 15.0–21.0), while the N660 group had a median score of 14.0 (IQR: 11.0–18.0). Therefore, there was a significant difference between the two groups (*p* = 0.007). For the GMP domain, the median score for the N327 group was 5.0 (IQR: 5.0–7.0) and for the N660 group was the same (5.0; IQR: 2.0–7.0). Thus, there was no significant difference when comparing the two groups (*p* = 0.367). When comparing the two groups for safety of use, there was a significant difference (*p* < 0.000). The median for the N327 group was 33.0 (IQR: 27.0–37.0) and the median for the N660 group was 5.0 (IQR: 1.0–9.0). Finally, for laboratory tests, the median for the N327 group was 0.0 (IQR: 0.0–3.0) and the median for the N660 group was also 0.0 (IQR: 0.0–4.0). When compared, there was no significant difference (*p* = 0.266; Fig. 4).

## Discussion

Implementation of the presented labeling scoring system could help guide professional prescribers and patients on which products sold in Brazil have the necessary information for safe prescription and use, given the wide range of products available and the regulations in force. Based on 45 items, the 105 selected products were evaluated and scored according to the weights assigned to them, which reflect the importance of this criterion for the proper prescription and use of the CBD-based products. Most of the evaluated products were rated as satisfactory (47), followed by 39 classified as not very satisfactory and 19 as very satisfactory. The lack of publicly available product information was the reason for most of the products falling into the two lower categories (satisfactory and not very satisfactory). The scores for the labeling quality criteria reflect the difficulties that prescribers and patients in Brazil face when prescribing/using CBD-based products, especially those from the N660. A yearbook mapping the cannabis market in Brazil estimated that in 2023, there were approximately 219,000 patients in the country who imported cannabis-based products, 114,000 who purchased through cultivation and distribution associations, and 97,000 who benefited from direct purchases from pharmacies/drugstores (Kaya-Mind 2023).

This study showed a wide variation in the labeling quality criteria of CBD-based products marketed in Brazil, both in the N327 and N660 groups, in the four domains evaluated. In the direct comparison between these two classes of products, there was a general difference between them. The N327 group had a significantly higher median score than the N660 group. Therefore, it can be concluded that products that have been notified with a temporary trade permit by the regulatory agency present quality information in a more accessible way than imported products, as it is a requirement that the package leaflet and standard packaging be made available on the official ANVISA website for products that have a trade permit.

In the prescription and safety of use domains, there was a significant difference in the final scores between the two product classes, i.e., the products in the N327 group performed better than those in the N660 group. No difference was found between the two classes of products in the areas of GMP and laboratory tests. This result was to be expected since, during the data collection, it was observed that neither the companies responsible for the imported products nor the companies with temporary trade permits in the country generally provide the data related to these two domains.

A significant difference was observed in the prescription domain when comparing products in the N327 group to those in the N660 group. The main factor contributing to this result was the requirement by the regulatory authority for package leaflets for those receiving temporary marketing authorizations, since most of the information about the product is found on the package leaflet, such as the correspondence between drops/mL, which is essential for prescribing. During the evaluation, it was observed that the products with the lowest scores for items related to the prescription requirements established by ANVISA were those in the N660 group. The Brazilian regulations (ANVISA, 2019) aim to establish the country's requirements for prescribing cannabis-based products. However, there are differences between N660 and N327. Ideally, relevant information should be provided to the patient at the time of prescription, including health risks, proof of safety and efficacy, possible adverse events, and precautions for use. Nonetheless, there are gaps in this information. There is still a significant difference between the two classes of products. The N327 products provide more information to consumers, although they do not present safety and efficacy data. Some authors highlight the importance of prescribing cannabis correctly, showing that communication, transparency about the treatment to the patient, and correct dispensing with proper guidance are essential information (Arnold et al. 2020; MacDonald & Adams., 2019). Thus, prescribing becomes easier and safer with more information available about the product. Prescribing a correctly titrated formulation with a known amount of active ingredients allows for more consistent therapeutic management and a better risk–benefit ratio (Citti et al. 2016; Romano & Hazekamp 2013; SIFAP, 2016).

In contemporary clinical practice, some providers consider using cannabis for conditions that are resistant to conventional treatment (i.e., epilepsy that is resistant to reference drugs). In contrast, others choose not to include cannabis in their therapeutic list due to the lack of robust evidence (Graham et al., 2023). Other providers are unaware of the therapeutic properties of cannabinoids, and there are still professionals who incorporate these products as adjuncts to integrative and complementary therapy. Thus, one of the most important aspects to be analyzed is the patient's responsiveness. For the best response, prescribers can adapt the treatment to their needs by changing the concentrations of the active ingredients, such as THC and CBD, of the products or their routes of administration (Beckett Wilson & Metcalf McGrath 2023). If the manufacturer refrains from providing information on the phytocannabinoids and terpenes present in the formulation, it is not possible to change this dose titration or product.

Safety of use was the area that showed the most significant difference between the N327 and N660 products. In other words, the products which received marketing authorization from the regulatory agency, even if temporary, had better scores on the items relating to the safety of use than those only authorized for import. Once again, the main factor contributing to this significant difference between the products is ANVISA's requirement for package leaflets for those which have received temporary marketing authorization. This is because most of the quality criteria relating to the safety of use domain are also found in the package leaflet, and those products without registration/temporary trade authorization are not required to present it.

The number of "CBD-containing" products available for sale, especially online, has grown exponentially in the USA. In addition, there is a lack of quality oversight and federal regulation of these products, which has led to an uncontrolled CBD market that, in turn, can result in negative outcomes, both concerning use and patient safety (Koturbash et al., 2020). A study conducted in the state of Mississippi (USA) of 25 commercially available CBD and THC-based products identified a marked variability between the actual CBD content and the declared amounts; only three were within ± 20% of their label declaration. In addition, the THC content of three products exceeded the legal limit of 0.3%, and four products were adulterated with synthetic cannabinoids, e.g., semi-synthetic ∆8-THC or HHC. The results of this study clearly demonstrated that most product labels did not accurately reflect the actual content and composition of the cannabinoids present and were, therefore, considered fraudulent, posing risks to the safety of use. The authors advocate the development of current GMP and their strict enforcement for cannabinoid products (Gurley et al. 2020). A South African study found that only three of the 40 products analyzed contained CBD at levels with a 90–110% margin of error. In addition, some of these products, which were supposed to be water-soluble, did not show a good aqueous solubility profile, and two products contained traces of THC (Mouton et al. 2024). This patchwork of laws and regulations surrounding the market for cannabis-based products in various countries around the world, as well as in the different states in the United States that authorize their use, has been widely discussed and pointed out by the scientific community as a risk to the safe use of these products (Britch et al., 2021; Pruyn et al. 2022).

There was no difference between the products from N327 for the GMP domain and those from N660. Most imported products come from the USA, where there is no federal oversight, and standards vary considerably between states. The FDA has approved the use of a few cannabis-based prescription products (Koturbash & MacKay 2020); otherwise, the FDA does not currently regulate CBD products as CBD is not considered a dietary supplement or food additive. However, it does issue safety announcements to warn the public of the potential risks of intoxication and that it is not just any product "good for everything" (Wagoner et al., 2021; Walker et al. 2020). The Brazilian regulatory agency considers cannabis-based products as medication, but even for N327 products, manufacturers have no legal obligation to provide data from laboratory tests involving user safety. As such, there was no difference between the groups, as much data was not provided.

Similarly, there was no difference between the two classes of products regarding the provision of laboratory tests. Even though some products did provide a laboratory test (31% of the N327 group and 45% of the N660 had a CoA), the majority did not. This may be directly linked to the fact that ANVISA does not require the information contained in the CoA to be made public, thereby leaving it up to the company to determine whether or not to make it available. The lack of transparency makes it challenging to obtain the desired information. It was also noted that, during the evaluation of the products in the N660 group, many manufacturers who made the CoA available had not carried out all the tests that were considered in this study (phytocannabinoid concentration, terpene characterization, presence of residues, contamination, microbiology, toxins, inorganic metals and foreign bodies, etc.), which may have influenced the scores of these products. Labeling claims (ingredients and their concentrations) are untrustworthy without a CoA. However, even if a product contains a CoA, it cannot always be trusted. Studies have demonstrated fraudulent labeling practices in the cannabis marketplace, including incomplete/incorrect label claims and false/incorrect CoA (Gurley et al. 2020; Mouton et al. 2024). Laboratory tests are extremely important for monitoring and controlling the quality of medications since phytocannabinoids can produce diverse effects and interfere with therapeutic management (Fischedick et al., 2010b). It is, therefore, imperative to implement control methods to guarantee the uniformity and quality of formulations (Omar et al., 2013) in order to provide users with a reliable product and ensure that providers are prescribing an accurate treatment. (Dussy et al. 2005; Fischedick et al. 2010a; Fischedick et al. 2010b).

The utilization of cannabis-based products is constrained not only in Brazil but also on a global scale. A paucity of controlled studies with an adequate sample size to yield clinically significant conclusions for diseases where potential benefits have been observed hampers the development of robust scientific evidence to support therapeutic indications. The masking of the placebo group, particularly in the case of products containing THC, represents a significant challenge for research centers engaged in the conduct of such studies. Furthermore, the quality control of currently available products is constrained by the absence of standardized testing protocols and methodologies designed to ensure the safety of users and prescribers. This is particularly evident in the absence of guidelines comparable to the pharmaceutical GMP. Brazil has established regulatory guidelines for GMPs for pharmaceuticals, including RDC 17/10 (Ministry of Health 2010), which has become the principal regulatory framework on this subject in the country, and RDC 658/22 (Ministry of Health 2022a, b), which presents the general guidelines for GMPs for pharmaceuticals.

Additionally, specific regulations have been established for cannabis-based products (Ministry of Health 2019). Subsequently, following a period of five years during which the regulation was in force, an extensive review was conducted. A report published by ANVISA indicated that the text of the regulation will be updated as soon as consultations and public hearings have been conducted to facilitate the implementation of the proposed changes. Introducing the revised regulatory framework is anticipated to result in a notable increase in the number of registered products within the country (ANVISA, 2023).

The regulation of cannabis for its health applications must be transparent, accountable, and publicly involved. These principles ensure that cannabis is used optimally, enhancing its therapeutic effects and mitigating adverse events (Gumilang et al. 2024). The more rigorous labeling quality criteria are established for these products, the greater the benefit to users. (Gurley et al. 2020; Johnson et al. 2022).

### Limitations of the study

The present study is subject to certain limitations in its development and analysis. The authors did not chemically analyze any products to confirm label claims (counter-evidence). Although this aspect is fundamental to complete the analysis of this problematic mechanism – ranging from a poor labeling system to a lack of CoA/laboratory testing requirements or third-party coherence – that generates total uncertainty about what is consumed, our study focused on evaluating the quality of products based on labeling information. Due to the considerable number of N660 products, not all of them were subjected to evaluation. The principal products available on the most frequently visited online marketplaces in the country were selected for analysis. The packaging of the products was not evaluated in situ; instead, the graphic representation published on the websites was assessed. A further limitation was the lack of response from most manufacturers to our email queries. This lack of response may have affected the scoring of the products, even if they did possess the requisite information. The evaluation of products imported from the USA was further constrained by the necessity to comply with state-specific regulations.

## Conclusion

This study found that most CBD-based products marketed in Brazil have a labeling quality classified as satisfactory, followed by not very satisfactory then very satisfactory, according to the established quality score. The products in group N327, which have notification and a temporary marketing permit in Brazil, presented the quality information in a more accessible way than the imported N660 products. Similarly, there was a significant difference between the groups of medications concerning the domains of prescription and safety of use; the products in the N327 group showed better results than those in the N660 group.

The data presented in this study will contribute to the urgent and necessary debate on the quality of labeling of CBD-based products widely marketed in Brazil. There is a need to consider the risks of consuming medications that do not present sufficient quality information, which may compromise the cost–benefit of their therapeutic application. In this sense, future studies should focus on analyzing the physicochemical and microbiological control of products currently marketed in the country to challenge the information presented on their labels and packaging. Furthermore, clinical trials should also be encouraged to demonstrate the safety and efficacy of these products for the variety of health conditions for which these drugs are prescribed. Finally, this study can inform the advancement of regulatory frameworks governing the therapeutic use of cannabis in Brazil. Specifically, it can contribute to regulating national production for scientific purposes and large-scale distribution through the Brazilian public and universal health system – the Unified Health System (SUS). In other words, as well as facilitating straightforward and cost-free access to a product with guaranteed quality and regulated by health bodies, it would enable the generation of scientific evidence, given the considerable number of individuals who utilize cannabis for therapeutic purposes in Brazil.

## Supplementary Information

Supplementary Material 1.

## Data Availability

No datasets were generated or analysed during the current study.
