# Supplementary material for: Evaluation of cannabidiol-based products in Brazil: how can current regulations influence their labeling quality?
Source: J Cannabis Res. 2025 Feb 22;7:12. doi: 10.1186/s42238-025-00270-2 (PMC11846405; doi:10.1186/s42238-025-00270-2)
Supplement: Supplementary file 1 — Supplementary Material 1. [file 42238_2025_270_MOESM1_ESM.docx]

**Supplementary Material**

**Table S1.** List of imported cannabis-based products covered by N660 and N327, which were selected for the study based on the selection criteria applied.

| **Products N660** | **Manufacturer** |
| --- | --- |
| 1 Pure CBD | Pure |
| 1Drop CBD | 1DROP |
| 57MED | 57MED |
| Abra-Cannabra | Sunny Skies CBD |
| ABX Oil Applicator Indica - 500mg | AbsoluteXtracts - ABX |
| Aceite Natursoy | Natursoy CBD |
| Active CBD | Discover Health Inc (Discover CBD) |
| Advantage For Life (AFL) CBD | Neurogan |
| Alese CBD | Korasana |
| Alivitta CBD | ALIVITTA LLC |
| Allandiol CBD | Biocase |
| Alliant CBD | Alliant |
| Alma CBD | CaniBrands Inc |
| Amaaya CBD | Amaaya |
| Antedotum CBD | Antedotum |
| Arkocapsulas Cannabis | Arkopharma Laboratorios |
| Aunt Zelda's - CBD | Aunt Zelda's TM |
| Aura Pharma Canibrands | CaniBrands Inc |
| Austral Hemp | Austral Hemp |
| Axon CBD | Axon Relief |
| Balance CBD Tincture | Balance CBD (AAXLL Supply Co LTD) |
| BAMF CBD Oil | Proof of Life CBD LLC |
| Barker Wellness CBD | Barker Wellness Co |
| Bedrocan CBD | Bedrocan |
| Beleaf Life's Oils CBD | Beleaf Life's Oil |
| Belle Avenue CBD | Belle Avenue |
| Beyond Botanicals | Beyond Botanicals LLC |
| Biokann CBD | Biokann CBD Healthcare |
| Bisaliv CBD | Thronus Medical INC |
| Black Pearl CBD | Black Pearl |
| Blue Raspberry CBDFX | CBDFX |
| Blue Ridge Hemp CBD | Blue Ridge Hemp |
| Blueharvest CBD | Blueharvest |
| Bluumlab Full Spectrum CBD | Bluumlab |
| Bontà Botanicals CBD | Bontà Botanicals Inc |
| Boston Hemp | Boston Hempire Inc |
| Botanique CBD | Botanique Premium Cannabidiol |
| Boticann CBD | Plena TM |
| Bud Life CBD | Bud Life |
| BudCare Wellness | Bud Temple |
| Bvo Indica | Santa Cruz Naturals |
| Cana Lab CBD | Oil Hanf |
| CandroPharm CBD | CandroPharm International |
| CaniBrands CBD | CaniBrands Inc |
| Cann Trust - CBD drops | Cann Trust |
| Canna Health Amsterdam CBD | Canna Health Amsterdam |
| Canna River | Canna River LLC |
| Cannab CBD (Designs for Health) | Designs For Health |
| Cannabidiol Life CBD Oil | Wholly Hemp INC |
| Cannabis Bakerhouse | Cannabis Bakerhouse |
| Cannabis Pharma Portugal | Cannabis Pharma Portugal |
| CannaBrasil | CannaBrasil |
| Cannaflower | Cannaflower |
| Cannajo CBD | Cannajo |
| Cannamedic CBD Oil | Cannamedic B.V. |
| Cannapresso CBD Oil - Tincture | Cannapresso CBD |
| CannaQIX 10 | Creso Pharm Switzerland GMBH |
| CanniFex CBD | CanniFex |
| Cannolex | Prime PBG Global |
| Carmen’s Medicinals Full Spectrum | Carmen’s Medicinals |
| Cannabinoids | Carmen's Medicinals |
| Carolina CBD | Carolina CBD Solutions |
| Cascadia Blooms Hemp Extract | Cascadia Blooms LLC |
| Casco Bay Hemp CBD | Casco Bay Hemp |
| CBD Calm | Kemin Industries Inc |
| CBD Emporium | CBD Emporium |
| CBD Genesis | CBD Genesis |
| CBD Konzentrat Gold 20% | Green Passion |
| CBD Lasca | Lasca Laboratorios |
| CBD Living Freeze | CBD Living |
| CBD Magic | CBD Magic INC |
| CBD OIL - Apollyon | Apollyon |
| CBD ÖL Mundtropfen | Hanfama GmbH |
| CBD Origin | Naturasor SL |
| CBD Pain Gel | USA Hemp CBD |
| CBD Queen | CBD Queen |
| CBD SKY | CBD SKY |
| CBD Vida - Nano-infused CBD | CBD Vida |
| CBDAlchemy Oil | CBDAlchemy |
| CBDBIOL | JGC COMPANY INC |
| CBDDENT | CBD Dent |
| CBDEX Care | OceanPharma LLC |
| CBDEx Oil | Cali Green Meds |
| CBDfx - CBD Balm Calming | CBDfx |
| CBDism | CBDism |
| CBDistillery | CBDistillery LTD |
| cbdMD Premium CBD | cbdMD LLC |
| CBFarma CBD | Korasana |
| Champlain - Sativa | Aphria |
| Charlotte Web Hemp Extract | CW Botanicals |
| Cheech's Stash CBD | Cheech's Stash |
| Cibadol Cannabidiol | Cibadol |
| Cibdex Hemp CBD | Complex Hemp Meds Px |
| Cibdol | Cibdol bv |
| CIDCAM CBD Aceite | CIDCAM Cannabis |
| Cili Swish CBD | CILI by design |
| Cilo Cybin CBD | Cilo Cybin |
| Clarify CBD Oil (CBDRX CBD Oil) | CBDRx LLC |
| Clever Leaves CBD | Clever Leaves 360 |
| Coba CBD | Coba |
| Comfort Body CBD | Comfort Body LLC |
| Connect | Sonder Fulfillment |
| Conscious CBD | Conscious by design |
| Country Farms Hemp Seeds Oil | Country Farms |
| Curatio Full Spectrum CBD | Curatio LTD |
| Curesupport | Curesupport |
| Custom CBD | Custom CBD |
| CV Sciences CBD | CV Sciences |
| Cycling Frog CBD | Cycling Frog |
| Cytogen | Global Health Brands LLC |
| Delta 8 CBD | Delta 8 Pharma Grade |
| Delta 8 Moon Rocks | Delta 8 Pharma Grade |
| Delta 8 PRO | Delta 8 PRO |
| Delta8 Diamond | Sun State Hemp |
| Diesel Hemp | Diesel Hemp |
| DiolPure CBD | DiolPure |
| Dixie Botanicals Hemp Oil | Hemp Meds Px |
| Dosecann CBD | Dosecann Cannabis Solutions |
| Dr. C Strains | Dr. C Strains |
| Dr. Formulated CBD | Garden of Life LLC |
| Dr. Monroe's CBD | Dr. Monroe's CBD Emporium |
| Dreamt | Ciencia Labs |
| DS Laboratories CBD | DS Healthcare Group Inc |
| Duda's Green Life CBD | 5 Day Private Label |
| Dynamic Medicinals CBD | Isonux LLC |
| EcoGen CDB | EcoGen Laboratories |
| ECS Care | ECS Therapeutics LLC |
| ELC CBD - Ease Labs | Ease Labs |
| Eleve CBD | Just Hemp CBD |
| Elite CBD - Full Spectrum | Elite Products International INC |
| Eliv CBD | Korasana |
| Elixinol Hemp Oil CBD | Elixinol |
| Elixir Organic CBD | Elixir Organic |
| Emerald CBD Oil | Emerald Health Therapeutics |
| Endoca Hemp Oil | Endoca |
| Enecta CBD | Enecta |
| Entourage CBD | The Native Hemp |
| Epidiolex | GW Pharmaceuticals |
| Epifractan CBD (2%-5%) | Medicplast S/A |
| EpileafCBD | WeLeafDNA |
| EpileafTHC | WeLeafDNA |
| ERVA CBD | ERVA Inc |
| Eurox Pharma | Eurox Group |
| EVA CBD | Anna Pure Hemp CBD |
| Evolve Nanoserum Hemp | Evolve Formulas LCC |
| Evona CBD | Hemp For Fitness LLC |
| EVR Hemp Oil CBD | EVR Premium Hemp Oil |
| FAB CBD | FAB NUTRITION |
| FarmaUSA Day/Night | Biota Biosciences LLC |
| Feel Good Health CBD | Feel Good Health |
| Fern Valley Farms | Fern Valley Farms |
| Fiore CBD | Fiore International Inc |
| FitoCBD - FitoFarma | Neurogan |
| Fitosil CBD | Fitosil |
| Five CBD Oil | Five CBD |
| Flextem Cannabis | Flextem Biopharma |
| Flor de María CBD | Flor de María |
| Flora Linda CBD | Flora Linda Botanicals |
| FlowerMed | FlowerMed LLC |
| Fluent CBD | Fluent Cannabis Care |
| FoliuMed CBD | FoliuMed |
| Forest Gold (CBD Fla) | CBD Fla |
| Foria Intimacy CBD | Eden Partners LLC |
| Formula A - Standarlized Canabidiol | Formula A TM |
| Formula Swiss - Full Spectrum CBD | Formula Swiss A.G |
| Fotmer CBD | Fotmer Life Sciences |
| Freely CBD | Freely Products |
| Full Spectrum Biologics | Full Spectrum Biologics |
| Funat CBD | Funat SAS |
| Funk Farms CBD | Funk Farms CBD Extracts |
| GEM CBD | GEM LLC |
| Glou CBD | Glou |
| Gold Label CBD 12% | Green Passion |
| Goldbar420 CBD | Goldbar420 |
| Golden CBD | Nano Golden CBD Plus |
| Great Ful CBD | Greatful |
| Green Care CBD | Green Care |
| Green Lake CBD | Green Lake LLC |
| Green Life Medicine CBD | Green Life Medicine |
| Green Relief CBD | Green Relief |
| Green Roads CBD | Green Roads |
| Greeneo CBD | GREENEO |
| Greenfield CBD | Greenfield |
| Greenmed - CBD | Greenmed |
| GreenPlus CBD Oil | TWA Brands GmbH |
| Greens Extract CBD | Greens Pharmaceutical Corp |
| GRN CBD | GRN |
| Hanftasia CBD | Hanftasia |
| Harbor Hemp | Harbor Hemp Company LLC |
| Harmony CBD | Harmony |
| Haurax CBD | Haurax |
| Healist Naturals CBD | Healist Advanced Naturals LLC |
| HealthCann CBD | HealthCann |
| HealthMeds CBD | Pure Life LLC |
| HealthyCann CBD | HealthyCann |
| Hemp & Azeite CBD - Bella Botanicals | Bella Botanicals |
| Hemp & Olive - Green Gorilla | Green Gorilla |
| Hemp Bio-vitality Oil | Dr. Botanicals |
| Hemp Care CBD Oil - Full Spectrum | Hemp Care Pharma |
| Hemp CBD (Bluebird Botanicals) | Bluebird Botanicals |
| Hemp Elixir - Sow eden | Sow Eden Organics |
| Hemp Extract | VESIsorb Pure Encapsulations |
| Hemp Farm CBD Oil | Hemp Farms do New York |
| Hemp Fusion - Phytocomplex | Hemp Fusion |
| Hemp Love Farms CBD | Hemp Love Farms |
| Hemp Private Labs CBD | Hemp Private Labs |
| Hemp Vegan | Hemp Vegan |
| HempClinic CBD | Hempclinic |
| HempFlex CBD - Green Care | Green Care |
| HempIndica CBD | Hemp Indica |
| Hemplucid CBD | HempLucid |
| Hempmeds Active Relief Roll-on | Hemp Meds Px |
| HempSmart CBD | hempSMART TM |
| Hemptouch - CBD Oil | Amber Hemptouch d.o.o. |
| Hempura CBD | Hempura |
| Hempzilla CBD | Hempzilla |
| Herbal Healers CBD | Herbal Healers |
| Herbdon CBD | Herbdon |
| Herbliz CBD | Herbliz Berlin |
| Herbstrong CBD | Sweetman Enterprises LT |
| HereNow CBD | HereNow |
| HOH CBD | HOH Root to Retail |
| Holland e Barret CBD | Holland & Barret |
| Holynol CBD | Holynol Labs LCC |
| Honest Botanical - CBD Oil | Canada Honest Botanical - CO |
| Honey Pot Cannabis - THC | Honey |
| Hopen CBD | Neurogan |
| Industrial Hemp Farms CBD | Industrial Hemp Farms |
| Infinite CBD | Infinite |
| Isoderm CBD | Isodiol |
| Isodiolex CBD (Isodiol) | Isodiol |
| Isospec CBD | Isospec Ltd |
| IUVO Broad Spectrum CBD | IUVO Therapeutics GmbH |
| JazzminCBD | Prime My Body |
| Jivameds CBD | Korasana |
| Jolli - Eternel Hemp | Eternel Hemp Farms |
| Just Hemp CBD | Just Hemp |
| Kalaya | Kalaya |
| Kalimediol CBD | Korasana |
| KannabidiOil | KannabidiOil |
| KannaSwiss - Full Spectrum | Organic Hemp |
| Extract | KannaSwiss |
| Kannaway CBD | Kannaway |
| Kanuf CBD | Kanuf |
| Kat's Naturals CBD | Kats Naturals LLC |
| Khiron - CBD | Khiron Life Sciences Corp |
| Kiara CBD | Kiara TM |
| Kingdom Harvest CBD | Kingdom Harvest |
| KM Relief tincture | KM Relief |
| Koba CBD | Healthy Grains S.A. |
| Koi CBD Oil | Koi CBD |
| Kopassion CBD | Kopassion |
| Korasana Full Spectrum CBD | Korasana |
| Lalana CBD | Dela-Nor LLC |
| Lamensdorf CBD | Alvit LCS Pharma |
| Landracer CBD | RAW Landracer CBD Solutions BV |
| Lazarus Naturals - CBD | Lazarus Naturals |
| LDN CBD | LDN CBD |
| Leaf CBD | Leafmed Care |
| Level Select CBD | Level Select TM |
| LGP Classic | Little Green Pharma |
| Liberi Cannabis | Gironatto LLC |
| Lifexpress CBD | Lifexpress |
| Lily CBD | Lily Health |
| Lord Jones CBD | Redwood Wellness LLC |
| Lucovitaal CBD | Lucovitaal |
| LyftED - Eternel Hemp | Eternel Hemp Farms |
| MAHARA CBD Oil | MAHARA CBD Group |
| Manitoba Harvest CBD | Manitoba Harvest |
| Mary's Elite CBD | Mary's Nutritionals |
| Medcan Australia CBD | Medcan Australia |
| MediCabilis CBD | Bod Australia |
| Medical Cannabis Cannassure | Cannassure |
| Medical Mary Full Spectrum CBD | Medical Mary LLC |
| MediPharm CBD | MediPharm Labs |
| Medix CBD | Medix CBD |
| Medkaya CBD | Reakiro Poland Sp |
| MedReleaf | MedRealef |
| Medropharm CBD | Medropharm GmbH |
| MEDTerra's CBD Oil | MEDTerra |
| Meraki CBD | Meraki |
| Meroflex | Grunelabs |
| MGC Pharma (CBD/THC) | MGC Pharma |
| MHL CBG Isolated | Mile High Labs International |
| MJ CBD | Delta 8 Mary Jane's CBD Dispensary |
| Monkey CBD | Balm Monkey Products |
| Moonlion CBD | Moonlion Hemp |
| Mother Holistic CBD | Mother Holistic LLC |
| Nabix | Urbanbox |
| Nano Hemp Tech Labs | Nano Hemp Tech Labs |
| Nano Mist Atomizer | Monarch Life Science LLC |
| NanoLab CBD | NanoLab Nutrition LLC |
| Nasadol CBD | Bio Spectrum Hemp |
| Naternal CBD | Naternal |
| Native Hemp Tincture | The Native Hemp |
| NatureCan Broad Spectrum CBD | Oil Naturecan LTD |
| Natureight CBD | Natureight |
| Nature's AID CBD | Nature's AID |
| Naturicious CBD | Naturicious France |
| Natyva CBD | Hammer Enterprises - Natyva Care |
| Naxiens CBD Oil | NATPHAR, LLC |
| Naysa CBD | Naysa Premium Grade |
| Neurogan CBD | Neurogan |
| New Age CBD Drops | New Age Hemp LLC |
| No Cap Hemp | No Cap Hemp Co |
| Nordic Oil CBD | Nordic Health Group |
| Nuleaf Naturals CBD Oil | Nuleaf Naturals LLC |
| Nutiva CBD Oil | Nutiva Nurture Vitality |
| Nutrient CBD | Nutrient CBD (Honest Globe) |
| Nutrify CBD | Nutrify Science |
| Odonto CBD | Moonlion Hemp |
| Ohemp CBD | ECS Therapeutics |
| Oliver's Harvest CBD | Oliver's Harvest |
| Onda CBD | Onda Wellness |
| One CBD | One Biosciences |
| One Lab CBD | One Lab CBD |
| Opulente CBD | Opulente Organics |
| Orange County CBD | Orange County UK |
| Organic CBD Oil | EcoGen Bioscience |
| Organica CBD | Organica CBD |
| Original Hemp | Arise Bioscience Inc |
| Otown CBD | Otown Hemp Co |
| Ouachita CBD | Ouachita Farms |
| Pachamama CBD | Pachamama LLC |
| Pacificool CBD | MRJ LLC |
| Pana Health | Panacea Life Sciences |
| Panaxia CBD/THC | Panaxia Pharmaceutical Industries Ltd |
| Panaxia THC | Panaxia Pharmaceutical Industries Ltd |
| Pangaia CBD | Pangaia (Elite Products International) |
| Pebble | Pebble Global Holdings |
| Peels CBD | Peels |
| Pharma Hemp CBD | PharmaHamp d.o.o. |
| PharmaCanna CBD | PharmaCanna |
| Pharmaoil CBD | Pharmaoil |
| Piping Rock Hemp Oil | Piping Rock |
| Plain Jane CBD | Plain Jane |
| Plant Based Labs CBD | Plant Based Labs |
| Plant Panda CBD | Plant Panda |
| PlantaDEA CBD | PlantaDEA |
| Plus CBD Oil | Plus CBD Oil |
| Polite CBD | Polite Hemp TM |
| Prima CBD Oil | The Uplifters' Prima PBC |
| Primitive Oils CBD | Primitive OilsTM |
| ProLife CBD | ProLife CBD |
| ProLife Hemp Oil CBD | ProLife Hemp |
| Promediol CBD | Swiss Therapeutic Solutions Promed Swiss S.A. |
| Proper Canna CBD | Proper Canna Naturals |
| Provacan CBD | Ciitech |
| PUCMED CBD | Florelar SAPUCMED |
| Pura Earth CBD | Avicanna Pura Earth |
| Pure Hemp Botanicals | Pure Hemp Botanicals |
| Pure Ratios CBD | Topical Path Pure Ratios |
| Pure Spectrum Cannabidiol | Pure Spectrum HQ |
| Pure Sun CBD | Pure Sunfarms |
| PureKana CBD | PureKana LTD |
| Purified CBD oil | Feel Good Health |
| PurMed CBD | PurMed Global TM |
| Purodiol CBD | Purodiol Limited UK |
| Quality CBD | Quality Cannabinoids |
| Quantic Herbs CBD | EndoCanna Health |
| Rapid CBD | Isodiol |
| Rare Cannabinoid | Rare Cannabinoid Company |
| Rational Hemp CBD | Mountain Oil Trading Inc |
| Reakiro CBD Oil | Reakiro Poland Sp |
| Real Scientific Hemp Oil (RSHO) CBD | Hemp Meds Px |
| Realize | Pure Global Inc |
| Redwood CBD | Redwood Reserve |
| Re-Leaf - Icon Hemp | Icon Hemp |
| Rethink CBD | Rethink |
| Reuni CBD Oil | Remederi USA LLC |
| Revida Labs CBD | Revida Labs TM |
| Revivid Hemp CBD | Revivid LLC |
| Royal CBD | Aceites Royal CBD |
| Sana Botanicals CBD | Sana Botanicals |
| Sana Hemp Juice | Hemp Juice Company BV |
| Sansal CBD | Sansal |
| Santeer CBD | Santeer TM |
| Saratoga CBD | Saratoga CBD Company |
| Satimed Phytocannabinoid | SatiMed UAB |
| Sativida CBD | Sativida |
| Second Century AG CBD | Second Century Ag, LLC |
| SELFe CBD | Self Evolution |
| Sensi CBD | Sensi Seeds |
| Sinceritas CBD Oil | Sinceritas JSC Subsidiary |
| Skin Restore | Green Care |
| Sol CBD | Sol CBD |
| Solverra CBD | Solverra |
| Sono CBD | Sono |
| Soulev CBD | Soulev |
| Southern CBD Solutions | Nashville CBD Solutions |
| Spectrum Cannabis | Canopy Growth |
| Spectrum Therapeutics | Spectrum Therapeutics |
| Speedy Naturals CBD | Speedy Naturals |
| Sphera CBD | Sphera Joy |
| Spirit Leaf CBD | Spirit Leaf |
| St. John's Nutrition - CBD Oil | St. John's Nutrition |
| Sunny Skies CBD | Sunny Skies CBD |
| Sunsoil CBD Oil | SUNSOIL |
| Supreme Kalm CBD | Supreme Kalm |
| Surterra Theragels CBD | Surterra Wellness |
| Sweet Lyfe Gummies | Sweet Lyfe |
| Swissextract CBD | Swissextract |
| Synchronicity CBD | Functional Remedies LLC |
| Syqe air herbs | Syqe Medical |
| T5K CBD - KM Relief | KM Relief |
| Tactical Reliaf CBD | Allied Corp |
| Tegra EUROLINE CBD | Korasana |
| Tegra Latam Line | FoliuMed |
| Tegra Proline CBD | Ciitech |
| Tegra USALINE CBD | Korasana |
| Terramed CBD | Rebotanicals Inc |
| The CBD Side | The CBD Side |
| The Lunatic CBD | MJ Packaging LLC |
| TheraCeed CBD | Columbia Care |
| Theraplant CBD | Theraplant |
| Tilray | Tilray Medical |
| Tinkun CBD | Tinkun Olam |
| Tinkun THC | Tinkun Olam |
| TM CBD | TM Pharmaceutical |
| Tropical CBD | Tropical CBD |
| Trulieve Truclear Concentrate | Trulieve |
| TudoLegal CBD | TudoLegal |
| Tweed Highlands Oral Spray | Delta 9 Cannabis |
| UB Super | UB Super |
| Ultimate Nutritional CBD | Softgels Ultimate Nutritional Inc |
| Ultrum CBD | Ultrum CBD |
| Upaya CBD | Upaya CBD |
| UpState Elevator CBD | UpState Elevator Supply Co |
| USAHemp CBD | USA Hemp CBD |
| Valens CBD | Valens CBD LLC |
| Valenss Wellness CBD | Neurogan |
| Valtellinamed CBD | Valtellinamed |
| Verdecann Aceite CBD | Verdecann |
| Verdemed CBD | Verdemed |
| Verdemed Sativyl | Verdemed |
| Verdi CBD Oil | Verdi SL |
| Veritas Farm Tincture | Veritas Farm |
| Verum Viridi CBD | Verum Viridi |
| Vida Biológica CBD | Vida Biológica |
| Vitadol CBD | Vitadol |
| VitalCBD | VitalCBD |
| Vitality CBD | Vitality CBD LTD |
| Vitox CBD | Vitox |
| Volcanic CBD | Sonder Fulfillment |
| Walden Extract | Walden |
| Warfighter CBD | Warfighter Hemp |
| Water Soluble CBD | American Shaman |
| Wild Orchard | Wild Orchard Co |
| Wisgro Hemp Farm CBD | Wisconsin Growing CO |
| Xannadiol CBD | Caillon Hamonet |
| Xativa CBD | iX Syrinx Pty Ltd |
| XK CBD | Exka Inc |
| Xula CBD | Xula |
| Yuluka Health CBD | Yuluka Health |
| ZAMZ CBD | Zamz |
| Zatural CBD | Zatural TM |
| Zipvit CBD | ZipVit Ltd |
| **Products N327** | **Manufacturer** |
| Canabidiol Aché | Aché Laboratórios Farmacêuticos |
| Canabidiol Active Pharmaceutica 20 mg/ml | Active Pharmaceutica Ltda |
| Canabidiol Aura Pharma | Aura Pharma S.A. |
| Canabidiol Belcher | Belcher Farmacêutica do Brasil Ltda |
| CannaBrasil | CannaBrasil |
| Canabidiol Collect | Collect Importação e Comércio Ltda |
| Canabidiol Ease Labs | Easelabs Laboratório Farmacêutico Ltda |
| Canabidiol Eurofarma | Eurofarma Laboratórios S.A. |
| Canabidiol Farmanguinhos | Fundação Oswaldo Cruz – FIOCRUZ |
| Canabidiol Greencare 23,75 mg/ml | Greencare Pharma Ltda |
| Canabidiol Herbarium | Herbarium Laboratório Botânico Ltda |
| Canabidiol Makrofarma | Makrofarma Química Farmacêutica Ltda |
| Canabidiol Mantecorp | Farmacosmed Indústria de Cosméticos e Medicamentos S.A |
| Canabidiol NuNature | Nunature Distribuição do Brasil Ltda |
| Canabidiol Prati-Donaduzzi | Prati Donaduzzi & Cia Ltda |
| Canabidiol Promediol | Promediol do Brasil Ltda |
| Canabidiol Verdemed | Verdemed Farmacêutica Ltda |
| Extrato de Cannabis Sativa Teuto | Laboratório Teuto Brasileiro S.A. |
| Extrato de Cannabis Sativa Cann 10 pharma | Cann 10 Pharma |
| Extrato de Cannabis Sativa Cannabr | Cannabr Distribuidora de Medicamentos |
| Extrato de Cannabis Sativa Greencare | Greencare Pharma |
| Extrato de Cannabis sativa Zion | Medpharma |
| Mevatyl | Ipsen |

**Table S2.** List of the 45 labeling quality criteria evaluated in the products to build their quality scores, their respective weights, and the domain in which the item is inserted.

| **Item** | **Domain** | **Weight** |
| --- | --- | --- |
| 1. Does it contain the product's trade name? | Prescription | 3 |
| 2. Does it list the qualitative composition of each active ingredient (phytocannabinoid)? | Prescription | 3 |
| 3. Is the concentration of each active ingredient (phytocannabinoid) indicated per unit of measurement on the packaging? | Prescription | 3 |
| 4. Does it include the name, address and telephone number of the company that produced the medicine? | Safety of use | 3 |
| 5. Does it state that it is a cannabis-based product? | Safety of use | 3 |
| 6. Is there a batch or tracking number? | Safety of use | 3 |
| 7. Does it show the date of manufacture of the final product? | Safety of use | 3 |
| 8. Is there an expiry date on the final product? | Safety of use | 3 |
| 9. Does it show the total amount of net weight or volume? | Prescription | 3 |
| 11. Are there criteria for matching the number of drops to 1 ml of the product? | Prescription | 3 |
| 12. Is there information on the possibility of poisoning and adverse effects? | Safety of use | 3 |
| 13. Is the product registered with a regulatory agency? | GMP | 3 |
| 14. Does the quality control (laboratory test - CoA) show the date the laboratory test was carried out and the product batch number reference? | GMP | 3 |
| 15. Are colony-forming unit (CFU) counts of fungi and aerobic bacteria and the absence of highly pathogenic bacteria described? | Laboratory tests | 3 |
| 16. Have solvent residues in the final product been characterized? | Laboratory tests | 3 |
| 17. Have possible contaminations by pesticides, herbicides and fungicides in the final product been characterized? | Laboratory tests | 3 |
| 18. Was the absence of aflatoxins or mycotoxins characterized in the quality control? | Laboratory tests | 3 |
| 19. Has the absence of inorganic metals (arsenic, cadmium, lead, nickel, copper, cobalt, chromium and manganese) been characterized? | Laboratory tests | 3 |
| 20. Was the absence of foreign bodies or impurities (chlorophyll and lipids) characterized in the final product? | Laboratory tests | 3 |
| 21. Is the product Good Manufacturing Practice (GMP) certified? | GMP | 2 |
| 22. Does it list the pharmaceutical form (oil, spray, capsules)? | Prescription | 2 |
| 23. Does it indicate the route of administration (oral, sublingual)? | Prescription | 2 |
| 24. How to use the product? | Safety of use | 2 |
| 25. Does it state that the product should be kept out of the reach of children? | Safety of use | 2 |
| 26. Is there risk information for pregnant and breastfeeding women? | Safety of use | 2 |
| 27. Is there a warning saying "While using the product, the patient should not drive vehicles or operate machinery or carry out activities that involve risks to themselves or others, as their ability and attention may be impaired"? | Safety of use | 2 |
| 28. Does it say: "Do not exceed the use indicated by the prescriber"? | Safety of use | 2 |
| 29. What are the main expected clinical effects? | Safety of use | 2 |
| 30. What are the main expected adverse effects? | Safety of use | 2 |
| 31. Does it contain the phrase "This product does not have efficacy and safety evaluated by the regulatory agency" or "This product does not have the complete clinical studies that prove its efficacy and safety" or "There are uncertainties regarding the long-term safety of the use of Cannabis products as medical therapy"? | Safety of use | 2 |
| 32. Does it include conservation precautions such as temperature range and storage conditions? | Safety of use | 2 |
| 33. Is there information on the diluent vehicle or excipients used in the product? | Prescription | 2 |
| 34. Does the product have dark or amber colored glass packaging? | GMP | 2 |
| 35. Does the product have millimeter pipettes? | Prescription | 2 |
| 36. Are the main terpenes found in the formulation listed? | Laboratory tests | 2 |
| 37. Have other phytocannabinoids such as cannabigerol (CBG), cannabidivarin (CBDV) or cannabichromene (CBC) been characterized in the final product? | Laboratory tests | 1 |
| 38. Is the presence of degradation products such as cannabinol (CBN) described? | Laboratory tests | 1 |
| 39. Was the quality control (laboratory test - CoA) carried out by an independent third party? | GMP | 1 |
| 40. Are there any clinical efficacy studies on the product? | GMP | 1 |
| 41. Is there a customer service number for the company holding the registration? | Safety of use | 1 |
| 42. Is there the name and address of the company holding the registration in Brazil or the importing company (distributor)? | Safety of use | 1 |
| 43. Is the name of the technical manager and their respective professional registration available? | Safety of use | 1 |
| 44. Does it say "For Sale on Prescription", "Can Only be Sold with Prescription Withheld" or similar? | Safety of use | 1 |
| 45. Do the physical and organoleptic characteristics of the product appear? | Safety of use | 1 |
